# Temporal and sleep stage‐dependent agreement in manual scoring of respiratory events

**DOI:** 10.1111/jsr.14391

**Published:** 2024-11-04

**Authors:** Minna Pitkänen, Henna Pitkänen, Rajdeep Kumar Nath, Sami Nikkonen, Samu Kainulainen, Henri Korkalainen, Kristín Anna Ólafsdóttir, Erna Sif Arnardottir, Sigridur Sigurdardottir, Thomas Penzel, Francesco Fanfulla, Ulla Anttalainen, Tarja Saaresranta, Ludger Grote, Jan Hedner, Richard Staats, Juha Töyräs, Timo Leppänen

**Affiliations:** ^1^ Department of Technical Physics University of Eastern Finland Kuopio Finland; ^2^ Diagnostic Imaging Centre Kuopio University Hospital Kuopio Finland; ^3^ VTT Technical Research Centre of Finland Ltd Kuopio Finland; ^4^ Reykjavik University Sleep Institute, School of Technology Reykjavik University Reykjavik Iceland; ^5^ Center of Sleep Medicine University Hospital Charité Berlin Berlin Germany; ^6^ Respiratory Function and Sleep Unit, Clinical Scientific Institutes Maugeri IRCCS Pavia and Montescano Italy; ^7^ Division of Medicine, Department of Pulmonary Diseases and Clinical Allergology Turku University Central Hospital, Turku, Finland, and Sleep Research Centre, University of Turku Turku Finland; ^8^ Center for Sleep and Wake Disorders, Institute of Medicine, Sahlgrenska Academy Gothenburg University Gothenburg Sweden; ^9^ Department of Pneumology, ISAMB, Faculty of Medicine University of Lisbon Lisbon Portugal; ^10^ School of Electrical Engineering and Computer Science The University of Queensland Brisbane Australia; ^11^ Science Service Center Kuopio University Hospital Kuopio Finland

**Keywords:** agreement, apnea, hypopnea, scoring

## Abstract

Obstructive sleep apnea diagnosis is based on the manual scoring of respiratory events. The agreement in the manual scoring of the respiratory events lacks an in‐depth investigation as most of the previous studies reported only the apnea–hypopnea index or overall agreement, and not temporal, second‐by‐second or event subtype agreement. We hypothesized the temporal and subtype agreement to be low because the event duration or subtypes are not generally considered in current clinical practice. The data comprised 50 polysomnography recordings scored by 10 experts. The respiratory event agreement between the scorers was calculated using kappa statistics in a second‐by‐second manner. Obstructive sleep apnea severity categories (no obstructive sleep apnea/mild/moderate/severe) were compared between scorers. The Fleiss' kappa value for binary (event/no event) respiratory event scorings was 0.32. When calculated separately within N1, N2, N3 and R, the Fleiss' kappa values were 0.12, 0.23, 0.22 and 0.23, respectively. Binary analysis conducted separately for the event subtypes showed the highest Fleiss' kappa for hypopneas to be 0.26. In 34% of the participants, the obstructive sleep apnea severity category was the same regardless of the scorer, whereas in the rest of the participants the category changed depending on the scorer. Our findings indicate that the agreement of manual scoring of respiratory events depends on the event type and sleep stage. The manual scoring has discrepancies, and these differences affect the obstructive sleep apnea diagnosis. This is an alarming finding, as ultimately these differences in the scorings affect treatment decisions.

## 1 INTRODUCTION

The gold‐standard for diagnosing obstructive sleep apnea (OSA) is polysomnography (PSG). PSG data are scored for respiratory events, i.e. apneas and hypopneas, and the OSA severity is estimated based on the number of events per hour of sleep (i.e. the apnea–hypopnea index [AHI]). In the current clinical practice, the scoring of the respiratory events and sleep stages is done according to the rules published by the American Academy of Sleep Medicine (AASM; Troester et al., 2023); however, this visual pattern recognition task is prone to subjective interpretation and human errors (Alvarez‐Estevez & Fernández‐Varela, 2020). In addition, despite several versions of these rules over the past decades, different scorers and sleep centres might have their own conventions or interpretations of the AASM rules (Kuna et al., 2013; Ruehland et al., 2009). This variability in the scoring practice results in differences in OSA diagnosis, which can lead to misdiagnosis or suboptimal treatment decisions (Kuna et al., 2013; Ruehland et al., 2009). Even though the inter‐scorer agreement in respiratory event scoring from PSG signals has been previously studied (Alvarez‐Estevez & Rijsman, 2022; Collop, 2002; Kuna et al., 2013; Magalang et al., 2013; Malhotra et al., 2013; Pittman et al., 2004; Punjabi et al., 2015; Rosenberg & Van Hout, 2014; Ruehland et al., 2023; Whitney et al., 1998), the reasons behind the differences between scorers and the effect of sleep stages on the scoring agreement have not been comprehensively investigated. Therefore, it is crucial to investigate the differences in the scoring in more detail to standardize and optimize diagnostic accuracy.

Previous studies have reported inter‐scorer agreements measured by kappa coefficients in the range of 0.46–0.88 for the identification of apneas or hypopneas or both (Alvarez‐Estevez & Rijsman, 2022; Pittman et al., 2004). Kappa statistic for the AHI has been reported to be as low as 0.24 (Collop, 2002), whereas intraclass correlation coefficients (ICCs) for the AHI have ranged between 0.61 and 0.99 (Alvarez‐Estevez & Rijsman, 2022; Kuna et al., 2013; Magalang et al., 2013; Malhotra et al., 2013; Pittman et al., 2004; Punjabi et al., 2015; Ruehland et al., 2023; Whitney et al., 1998). Moreover, respiratory event types can affect the agreement; the scoring of obstructive apneas has shown higher agreement than the scoring of central and mixed apneas (Kuna et al., 2013; Magalang et al., 2013; Rosenberg & Van Hout, 2014), and the scoring of apneas has often led to higher agreement than the scoring of hypopneas (Alvarez‐Estevez & Rijsman, 2022; Rosenberg & Van Hout, 2014), but not always (Magalang et al., 2013).

A previous study investigated the agreement in scoring of respiratory events in a temporal manner by taking into account the timing of the events (Alvarez‐Estevez & Rijsman, 2022). However, in that study, the analysis was limited to event versus no event comparison (Alvarez‐Estevez & Rijsman, 2022). To the best of our knowledge, the inter‐scorer agreement in respiratory event scoring within different sleep stages has also not been previously done. Therefore, we investigated the temporal, i.e. second‐by‐second, agreement in the respiratory event scoring, and identified the respiratory event types and sleep stages with the most significant disagreement between scorers. Based on previous studies (Kuna et al., 2013; Magalang et al., 2013; Rosenberg & Van Hout, 2014), we hypothesized that the agreement in the scoring of obstructive apneas is higher than that of hypopneas, central and mixed apneas. Furthermore, we hypothesized that the temporal agreement would be low between the scorers as the scoring of the exact event duration or start and end times are not used for clinical scoring and non‐temporal analysis will mask much of the disagreement found for individual event scoring.

## 2 METHODS

### 2.1 Population

The study population (*n* = 50) consisted of healthy individuals and participants with an increased risk for sleep disorders including OSA, restless legs syndrome (RLS) or insomnia. The risk assessment was conducted by screening the participants using the STOP‐BANG questionnaire (Chung et al., 2012), the Insomnia Severity Index (Morin et al., 2011), and the International Restless Legs Syndrome Study Group Questionnaire (The International Restless Legs Syndrome Study Group, 2003). The demographic information of the population is presented in Table 1 and in more detail in our previous works (Nikkonen et al., 2024; Pitkänen et al., 2024). The inter‐scorer agreements in sleep stage scoring and arousal scoring in this dataset have been published earlier (Nikkonen et al., 2024; Pitkänen et al., 2024).

**TABLE 1:** Demographic information on the participants.

	*n*
Total number of participants	50
Male	29
Female	21
With OSA risk	29
With insomnia risk	17
With RLS risk	11
Healthy participants	9

	Mean (SD)
Age (years)	42.9 (13.7)
BMI (kg m^−2^)	27.3 (5.8)

BMI, body mass index; *n*, number of participants; OSA, obstructive sleep apnea; RLS, restless legs syndrome; SD, standard deviation.

### 2.2 Recordings and scoring

The PSG studies were conducted in 2021 using Nox A1 devices (Nox Medical, Reykjavik, Iceland). The PSG setup included electroencephalogram (C3, C4, F3, F4, O1, O2, M1, M2), electrocardiogram, electrooculogram, chin and leg electromyogram, nasal pressure, respiratory effort, oxygen saturation, pulse, and body position measurements. The PSG was set at Reykjavik University Sleep Institute after which the participants left and slept at their homes. The study was approved by the National Bioethics Committee of Iceland (21‐070, 16/3/2021) and all participants gave a written informed consent.

All 50 PSG recordings were scored by 10 expert scorers from seven different sleep centres. The PSG studies were scored for sleep stages, respiratory events, desaturations, and arousals according to the guidelines in AASM manual version 2.6 (Berry et al., 2020) using the recommended rules, with the exception that apneas were scored using the nasal pressure signal instead of oronasal thermistor. The scoring was done using Noxturnal software, Research Version 6.1.0.30257 (Nox Medical, Reykjavik, Iceland). To replicate everyday clinical practice, the scorers run the automatic PSG and Respiratory Analysis in the scoring software before manually scoring and correcting the events. In addition, the scorers were given the option to score events as “uncertain apneas” (0.04% of all respiratory events) or “uncertain hypopneas” (1.05% of all respiratory events) if unsure. Scorer pairs 1&2, 4&5 and 6&7 were from the same sleep centres.

### 2.3 Data preparation

The recordings were trimmed by removing all epochs where all scorers were not scoring sleep or wake. This was done to reduce the effect of excess wake in the beginning and end of the recordings, but also to exclude epochs marked as invalid by any scorer. Respiratory events shorter than 10 s (0.4% of the scored events) and starting in wake epochs were excluded (Berry et al., 2020; Troester et al., 2023). The following analysis was conducted separately for scorer‐specific sleep stages and for majority scored sleep stages. Majority sleep stages were calculated for each participant based on the most scored sleep stage in each epoch, similar to a previous study (Nikkonen et al., 2024). The sleep epoch linked to an event was the epoch during which the event started.

### 2.4 Event numbers and durations

The number and median duration of events were calculated. In addition, respiratory event densities in the sleep stages, i.e. the average number of scored events in an epoch by one scorer, were calculated. In the case of the scorer‐specific sleep stages, the number of all scored respiratory events in each scorer‐specific sleep stage was divided by the number of all given sleep stage epochs to get the density of events (Pitkänen et al., 2024). For the majority sleep stages, the number of all scored respiratory events in each majority sleep stage was divided by 10 times the number of all given majority sleep stage epochs (Pitkänen et al., 2024).

### 2.5 Second‐by‐second analysis

The agreement analysis was conducted in a temporal, second‐by‐second manner, in several ways: (1) binary classification of each second either as event or no event; (2) classification of each second either as no event, apnea or hypopnea; (3) event subtype classification of each second either as no event, obstructive apnea, central apnea, mixed apnea, hypopnea associated with desaturation only, hypopnea associated with an arousal only, or hypopnea associated with both desaturation and arousal; (4) binary (event versus no event) classification of each second in different sleep stages; and (5) binary classification of each second either as a specific event subtype (e.g. obstructive apnea) or no event (all other events were considered as no event). Hypopnea subtypes were formed retrospectively (after scoring of events by the scorers) as follows: hypopneas were considered to be associated with desaturation if desaturation occurred during the hypopnea or within 15 s after the hypopnea ended, whereas hypopneas were considered to be associated with an arousal if the arousal occurred during the hypopnea or within 10 s after the hypopnea ended. Several hypopnea events could be associated with the same desaturation event or arousal.

Moreover, a majority respiratory event score was formed. A second in the majority respiratory event score was set as an event if five or more scorers had scored an event during that second. In the case of a tie, the event with the highest priority was chosen. The priority order was: hypopnea associated with desaturation only, hypopnea associated with an arousal only, obstructive apnea, hypopnea associated with both desaturation and arousal, central apnea, mixed apnea, and apnea without type or labelled as “uncertain.” The order was based on the prevalence of the events, from the highest to the lowest.

### 2.6 Cluster analysis

To compare the start times and end times of respiratory events between scorers, each recording was divided into event clusters similar to a previous study (Pitkänen et al., 2024). A cluster comprised events that were scored around the same time in the recordings. The cluster start time was the onset of the first event scored by any scorer after a period of no events, and the cluster ended at the termination of the last event by any scorer before a period of no events. A cluster might have consisted of only one event or several events. If a scorer had scored several events in a cluster, the start time was the onset of the first event and the end time was the time of termination of the last event of that scorer. The pairwise accuracies in the clusters were calculated as the duration where the scorers agreed divided by the duration of the whole cluster.

### 2.7 Agreement and statistical analysis

Agreement in the different PSG parameters, such as total sleep time (TST), AHI, apnea index (AI), obstructive apnea index (OAI), central apnea index (CAI), mixed apnea index (MAI) and hypopnea index (HI), between the scorers was assessed using ICC(A,1), i.e. the degree of absolute agreement among scorers (McGraw & Wong, 1996). The OSA severity categories were formed based on the traditional AHI thresholds (no OSA: AHI < 5 events per hr; mild: 5 events per hr ≤ AHI < 15 events per hr; moderate: 15 events per hr ≤ AHI < 30 events per hr; severe: AHI ≥ 30 events per hr; American Academy of Sleep Medicine Task Force, 1999). The agreements in classifying participants to different OSA severity categories between the scorers and the second‐by‐second agreement of events were quantified using the kappa statistics and related observed agreement between scorers (*p*_*o*_). Cohen's kappa (Cohen, 1960) was used for quantifying pair‐wise agreement, and Fleiss' kappa (Fleiss, 1971) was used for multi‐scorer agreement. Pairwise comparisons were conducted between each scorer pair, as well as between each scorer and the majority score. Kappa statistics were calculated separately for the TST, scorer‐specific sleep stages, and majority sleep stages. For the agreement in different sleep stages, both the sleep stage and the event needed to be the same for agreement. A chi‐squared test was performed for the distribution of the number of participants in the different OSA severity categories based on the AHI values, with a significance level of 0.05. Moreover, the percentage of participants whose OSA severity category differed in at least two scorings was calculated.

## 3 RESULTS

### 3.1 Number and duration of respiratory events

**FIGURE 1:**
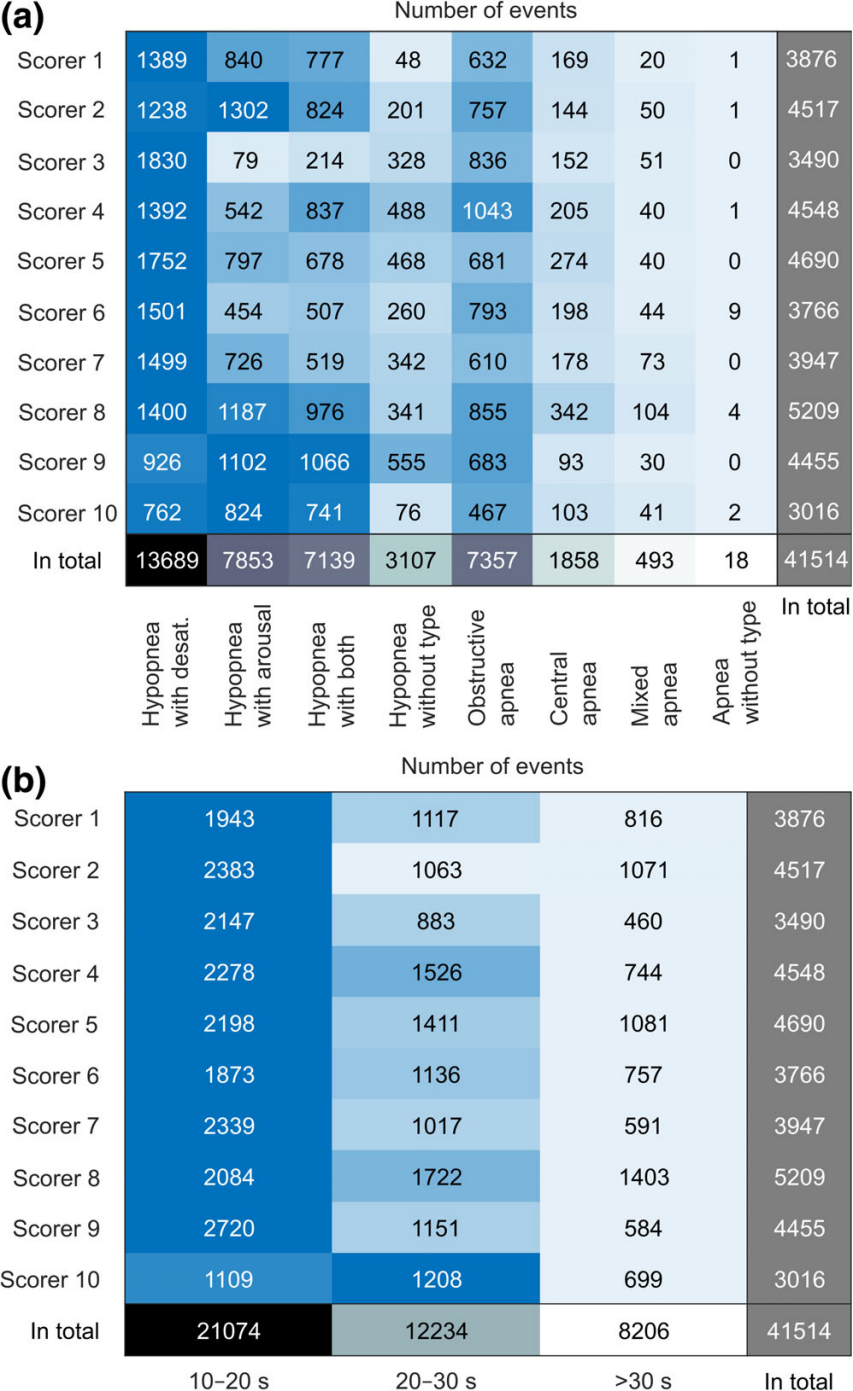
The number of scored events based on (a) their type and (b) duration. The numbers are colour‐coded so that the higher the number, the darker the colour (scorer‐wise).

**FIGURE 2:**
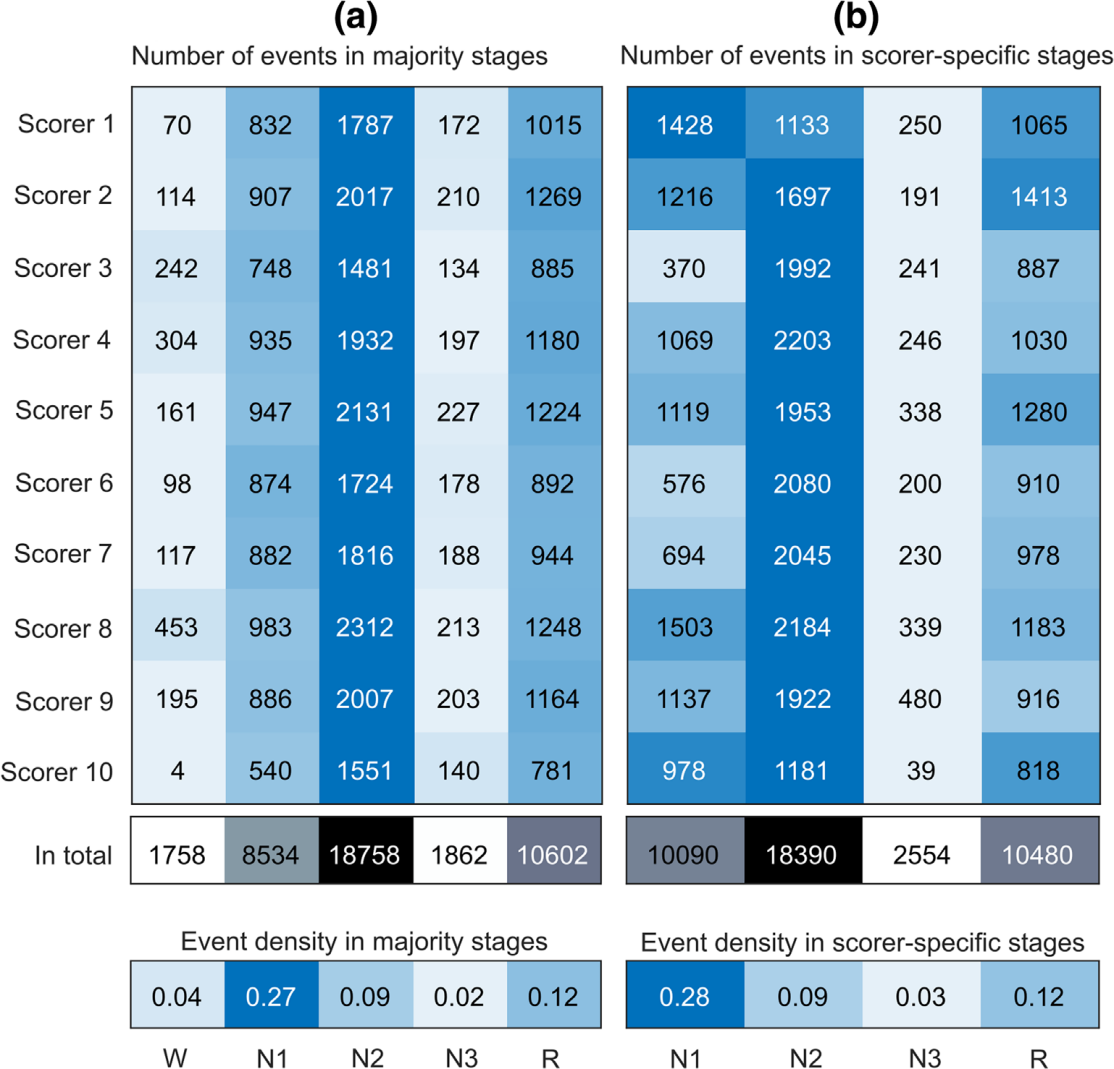
Number of the respiratory events scored by the 10 expert scorers (a) in the majority score‐based sleep stages and (b) in the scorer‐specific stages (excluding wake). In addition, below the numbers are the event densities in the sleep stages, i.e. the average number of scored events in an epoch by one scorer. The numbers are colour‐coded so that the higher the number, the darker the colour (scorer‐wise). The events occurring in scorer‐specific wake epochs were not included in the agreement analysis. The events occurring in majority score‐based wake epochs are shown in (a) so that the total number of events in all figures would be the same. N1, non‐rapid eye movement sleep stage 1; N2, non‐rapid eye movement sleep stage 2; N3, non‐rapid eye movement sleep Stage 3; R, rapid eye movement sleep stage; W, wake.

The most often scored event type was hypopnea associated with oxygen desaturation (Figure 1). The event density was highest in N1, followed by stages R, N2, wake and N3 (majority score) (Figure 2). Most often, the duration of a scored respiratory event was between 10 and 20 s (Figure 1). However, all scorers scored events less than 10 s (excluded from the analysis) and longer than 30 s.

### 3.2 Agreement in the severity indices

**FIGURE 3:**
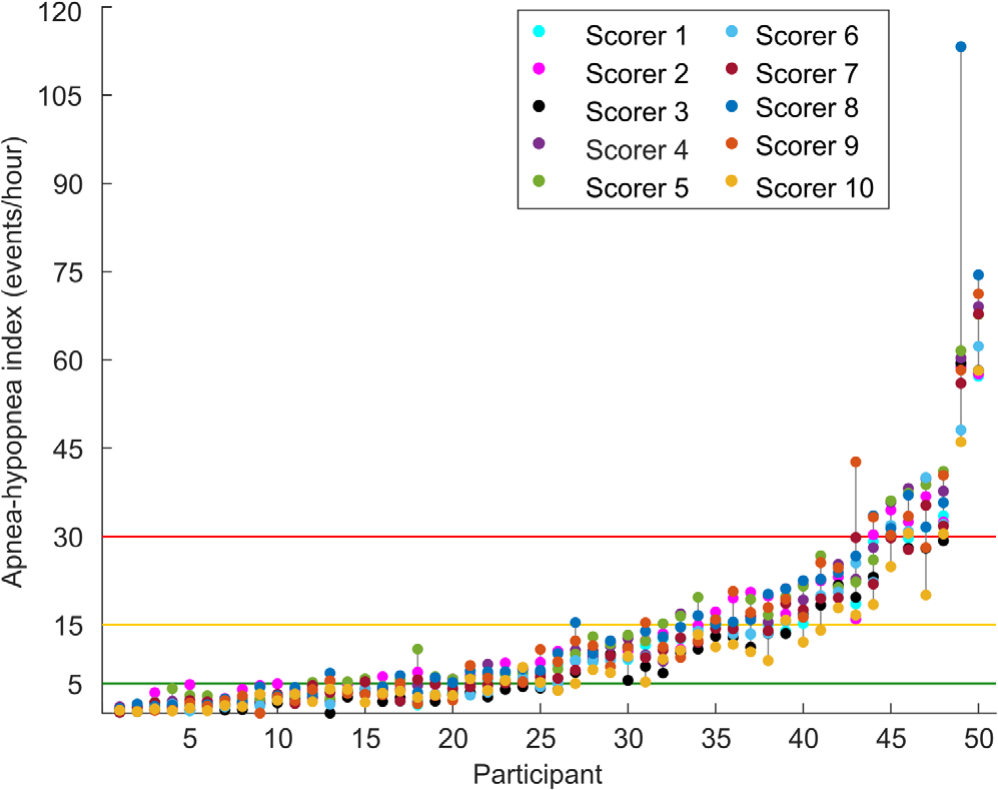
Apnea–hypopnea indices (events per hr [AHI]) of all 50 participants scored manually by all 10 expert scorers. Indices of the same participant are connected to each other with a vertical line. The horizontal lines represent the thresholds of 5, 15 and 30 events per hr. The participants are sorted based on their average index from the lowest to the highest.

**FIGURE 4:**
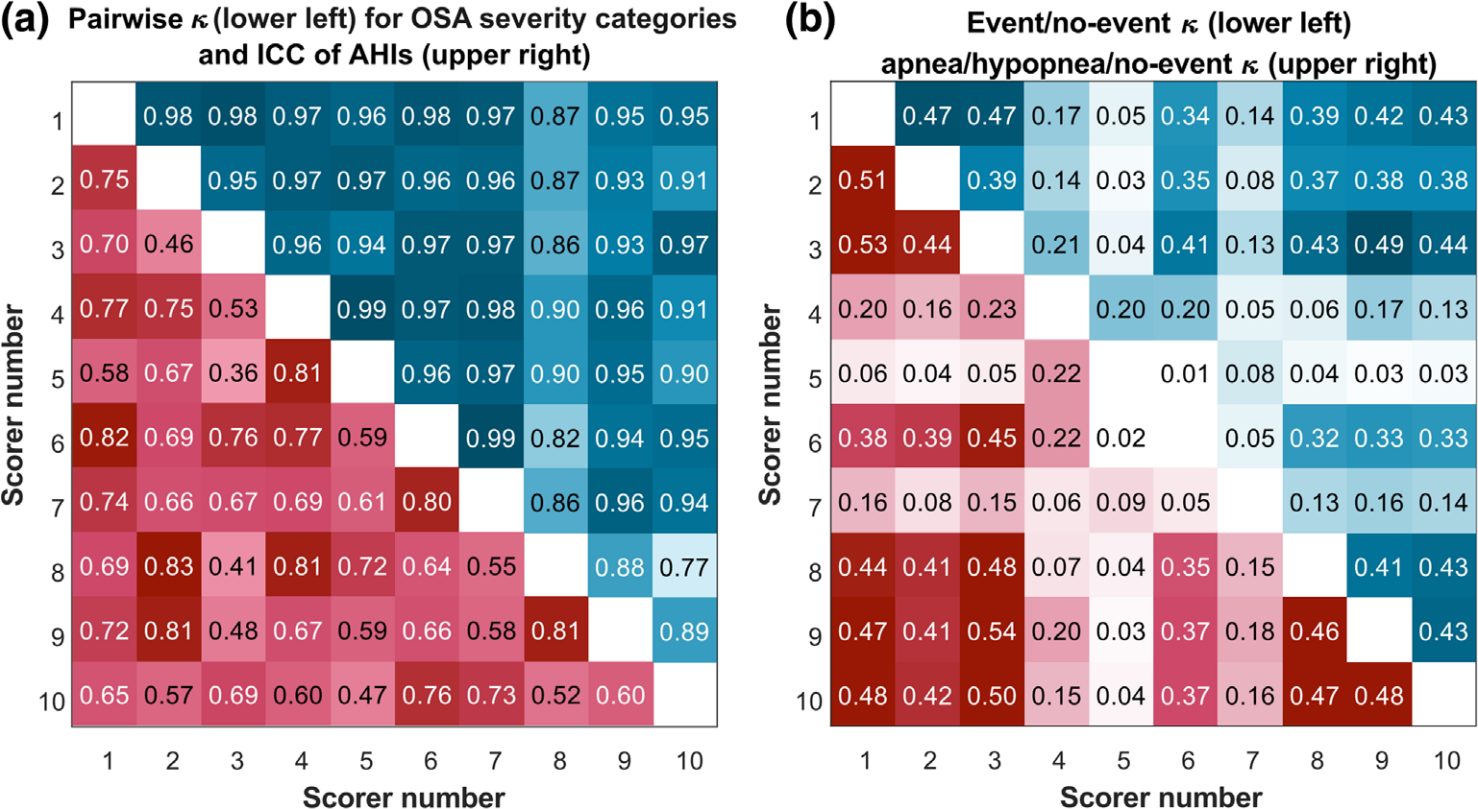
(a) Pairwise Cohen's kappa (κ) values for obstructive sleep apnea (OSA) severity categories (no OSA, mild, moderate and severe OSA) and intra‐class correlation coefficients (ICCs) of apnea–hypopnea indices (AHIs) between the 10 scorers for the 50 participants. (b) Second‐by‐second pairwise Cohen's κ for the event/no event scorings and apnea/hypopnea/no event scorings between the 10 expert scorers for the 50 participants. The numbers are colour‐coded so that the higher the number, the darker the colour.

We found high variability in the OSA severity indices between the scorers (Figure 3; Table 2). The highest ICC was found for AI, and the lowest for CAI (Table 2). Pairwise ICCs of AHIs ranged between 0.77 and 0.99 (Figure 4). The distribution of the number of participants in different OSA severity categories based on the AHI values did not significantly differ between the scorers (*p* = 0.954). However, in only 34% of the participants the OSA severity category stayed the same among the scorings, whereas in the rest of the participants at least one scoring differed so that the category changed (Figure 3). The percentage of participants classified to the no‐OSA category varied from 26% to 52% depending on the scorer. Similarly, the percentages of participants in the other severity categories varied significantly between the scorers: from 26% to 38% for the mild OSA category; from 12% to 24% for the moderate OSA category; and from 6% to 14% for the severe OSA category.

**TABLE 2:** Median (IQR) and ICC (95% CI) for the severity indices and TST.

Index	Median (IQR)	ICC (95% CI)
AHI (1 per hr)	7.2 (3.2–16.5)	0.93 (0.90–0.96)
AI (1 per hr)	0.8 (0.2–2.4)	0.95 (0.93–0.97)
OAI (1 per hr)	0.2 (0.0–1.5)	0.94 (0.92–0.96)
CAI (1 per hr)	0.2 (0.0–0.7)	0.63 (0.52–0.73)
MAI (1 per hr)	0.0 (0.0–0.0)	0.72 (0.64–0.81)
HI (1 per hr)	6.1 (2.4–14.0)	0.86 (0.80–0.91)
TST (hr)	6.8 (6.2–7.8)	0.96 (0.93–0.98)

AHI, apnea–hypopnea index; AI, apnea index; CAI, central apnea index; CI, confidence interval; HI, hypopnea index; ICC, intraclass correlation coefficient; IQR, interquartile range; MAI, mixed apnea index; OAI, obstructive apnea index; TST, total sleep time.

The Fleiss' kappa for classifying participants in different OSA severity categories was 0.66 (*p*_*o*_ = 0.76). The pairwise agreement between the scorers related to the severity classification of OSA showed significant variation with Cohen's kappa values ranging between 0.36 and 0.83 (*p*_*o*_ = 0.54–0.88; Figure 4). The ICC of the severity categories was 0.88 (95% confidence interval: 0.83–0.93). However, 22% of the AHIs were within ± 1 event per hr around the threshold values (i.e. AHI value was 4–6, 14–16 or 29–31 events per hr).

### 3.3 Second‐by‐second agreement

The second‐by‐second Fleiss' kappa between the scorers was 0.32 at maximum (Table 3). When calculated separately for the sleep stages, the Fleiss' kappa was the highest for stage R and the lowest for stage N1 (Table 3). The pairwise Cohen's kappa for binary classification between the scorers was 0.54 at maximum but as low as 0.02 for some scorer pairs (Figure 4). When comparing each scorer with the majority score, Cohen's kappa ranged between 0.07 and 0.55 depending on the scorer and classification type.

**TABLE 3:** Fleiss' kappa for the second‐by‐second classifications.

Event type classification	Fleiss' kappa
Event/no event (binary)	0.32 (*p*_*o*_ = 0.94)
Apnea/hypopnea/no event	0.29 (*p*_*o*_ = 0.94)
Apnea/no event	0.23 (*p*_*o*_ = 0.99)
Hypopnea/no event	0.26 (*p*_*o*_ = 0.95)
All event types	0.22 (*p*_*o*_ = 0.94)
Obstructive apnea/no event	0.21 (*p*_*o*_ = 0.99)
Central apnea/no event	0.12 (*p*_*o*_ = 1.00)
Mixed apnea/no event	0.05 (*p*_*o*_ = 1.00)
Hypopnea with desaturation only/no event	0.17 (*p*_*o*_ = 0.98)
Hypopnea with arousal only/no event	0.09 (*p*_*o*_ = 0.98)
Hypopnea with desaturation and arousal/no event	0.11 (*p*_*o*_ = 0.98)
Event in scorer‐specific sleep stages	0.24 (*p*_*o*_ = 0.93)
Event in scorer‐specific N1 (binary)/no event	0.11 (*p*_*o*_ = 0.98)
Event in scorer‐specific N2 (binary)/no event	0.19 (*p*_*o*_ = 0.97)
Event in scorer‐specific N3 (binary)/no event	0.15 (*p*_*o*_ = 1.00)
Event in scorer‐specific R (binary)/no event	0.21 (*p*_*o*_ = 0.98)
Event in majority‐specific sleep stages	0.26 (*p*_*o*_ = 0.94)
Event in majority scored N1 (binary)/no event	0.12 (*p*_*o*_ = 0.99)
Event in majority scored N2 (binary)/no event	0.23 (*p*_*o*_ = 0.97)
Event in majority scored N3 (binary)/no event	0.22 (*p*_*o*_ = 1.00)
Event in majority scored R (binary)/no event	0.23 (*p*_*o*_ = 0.98)

N1–N3, non‐rapid eye movement stages 1–3; *p*_*o*_, observed agreement; R, rapid eye movement stage.

### 3.4 Event clusters

**FIGURE 5:**
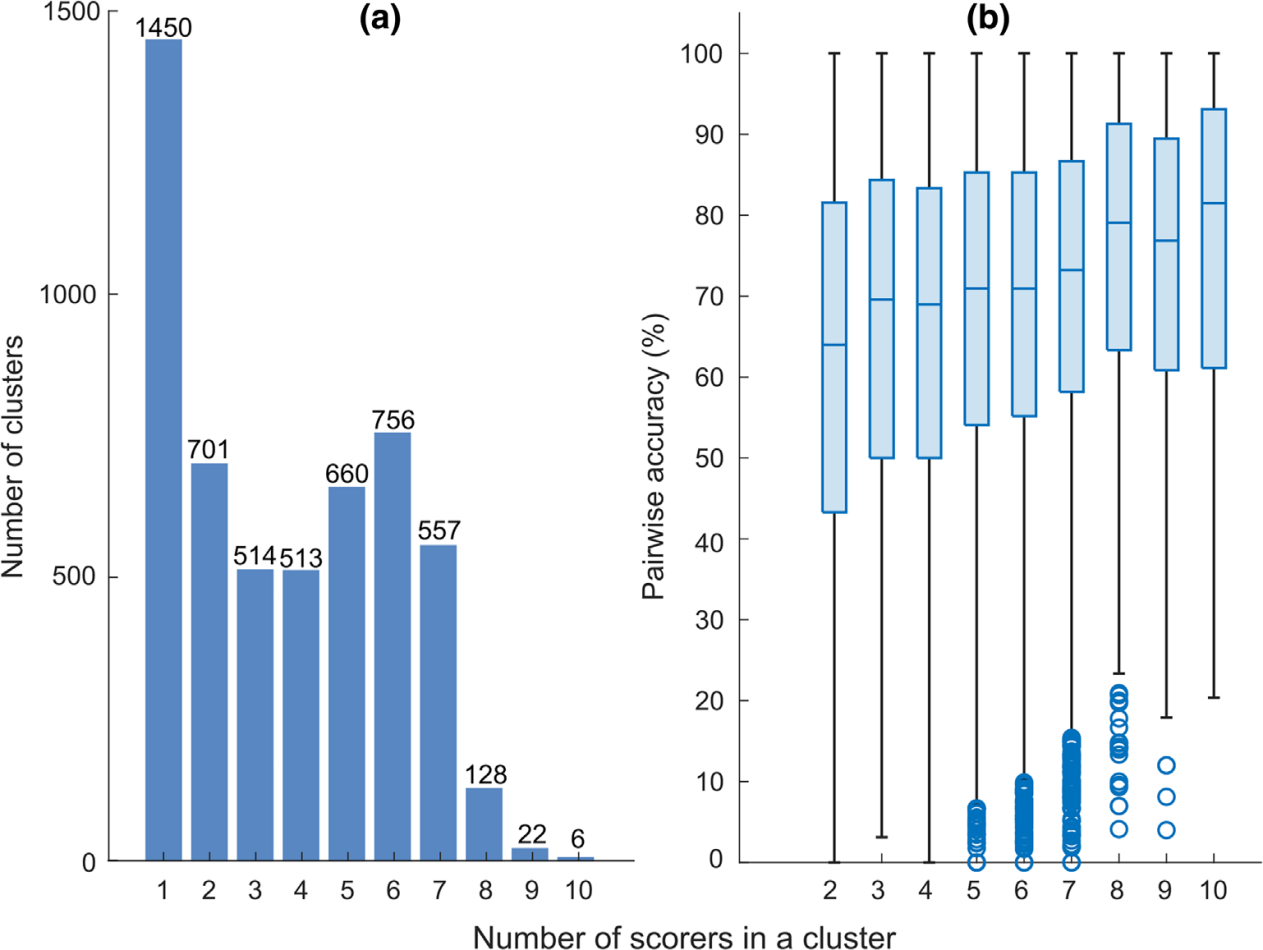
(a) The number of clusters based on the number of scorers in the clusters. (b) Pairwise accuracies, i.e. the durations inside the clusters during which the scorers agreed that there is an event or there is no event divided by the length of the whole cluster. Medians are shown as lines inside the boxes, the lower and upper quartiles as the bottom and top edges of the box, respectively, the maximum and minimum data values that are not outliers as whiskers, and the outliers (more than 1.5 times the interquartile range away from the bottom or top of the box) as circles.

There were 5307 respiratory event clusters. The median number of scored respiratory events in a cluster was 4 (interquartile range [IQR] = 1–6) and the median number of scorers in a cluster was 3 (IQR = 1–6). The majority respiratory event agreement, i.e. at least five scorers scoring respiratory events in a cluster, was reached in less than half (*n* = 2129, 40%) of the clusters (Figure 5a). In most of the clusters (*n* = 4485, 85% of the clusters), the scorers had scored a maximum of one respiratory event. The median cluster duration was 30 s (IQR = 21–47 s).

The median pairwise absolute agreement of the respiratory events in the clusters, i.e. the durations in the clusters during which scorers agreed divided by the length of the cluster, was 72% (IQR = 56–86%). The median agreement ranged between 64% and 81% when the clusters were grouped based on the number of scorers scoring respiratory events in the cluster (Figure 5b). Furthermore, the median event start time and end time differences between the scorers were 1 s (IQR = 0–4 s) and 6 s (IQR = 2–13 s), respectively.

## 4 DISCUSSION

In this study, we investigated the agreement in respiratory event scoring between multiple scorers, and found that the temporal, i.e. second‐by‐second, agreement was only fair. The agreement depended on the sleep stage and event type, being the highest in rapid eye movement (REM) sleep and with hypopneas. The ICCs of the severity indices, such as the AHI, indicated moderate agreement, and these findings are in line with previous studies (Alvarez‐Estevez & Rijsman, 2022; Kuna et al., 2013; Magalang et al., 2013; Malhotra et al., 2013; Pittman et al., 2004; Punjabi et al., 2015; Ruehland et al., 2023; Whitney et al., 1998).

The event type affected the temporal agreement. The findings of the study confirmed our hypothesis that there is a stronger agreement in the scoring of obstructive apneas than central and mixed apneas. Similar results have been observed in previous studies (Kuna et al., 2013; Magalang et al., 2013; Rosenberg & Van Hout, 2014). In our study, obstructive apneas were more frequently scored than the other apnea types, which can lead to higher kappa values. In addition, Fleiss' kappa was slightly higher for hypopneas than apneas, whereas the ICC for AI was higher than for HI. Thus, our results indicate that the agreement in the number of apneas is higher than the agreement in the number of hypopneas, whereas the agreement in the temporal aspects is higher for hypopneas than for apneas. Similar discrepancies in the agreement have also been found in previous studies; some studies indicating higher agreement for apneas than for hypopneas and vice versa (Alvarez‐Estevez & Rijsman, 2022; Kuna et al., 2013; Magalang et al., 2013; Rosenberg & Van Hout, 2014). These results may also be explained by the higher number and longer duration of hypopnea events compared with apnea events. In addition, we observed that the scorers had a higher agreement in hypopneas associated with desaturation than in hypopneas associated with an arousal. Again, the result might be affected by the frequency of the event type or uncertainties in arousal scoring. These results are consistent with a previous study that found a higher ICC for AHI when desaturation in comparison to arousal was required for scoring of events (Whitney et al., 1998).

The second‐by‐second agreement was the highest in R stage and the lowest in N1. To the best of our knowledge, the respiratory event scoring agreement in different sleep stages has not been previously investigated. It has been found that apneas are longer in stage R than in non‐rapid eye movement (NREM) sleep (Findley et al., 1985; Koo & Mansour, 2014; Nakayama et al., 2019), which could increase the temporal agreement in R stage. Similarly, shorter events during N1 sleep could explain the lower kappa values. However, the respiratory event agreement in different sleep stages is dependent on the sleep stage scoring in addition to the respiratory event scoring, and a previous study on sleep stage scoring agreement within the present dataset found the highest agreement in R stage and the lowest in N1 (Nikkonen et al., 2024), which could explain our results. Moreover, the respiratory events fragment the sleep especially in participants with severe sleep apnea and during N1 sleep. Therefore, sleep stage scoring is challenging due to the several sleep stage transitions, and some respiratory events may be missed if the epoch is scored as wake due to the current sleep staging rules. A more detailed investigation is required to explain the reason for the differences in event scoring agreement between sleep stages.

The temporal agreement of respiratory event scoring in this study was fair, and lower than previously reported for respiratory events (Alvarez‐Estevez & Rijsman, 2022; Pittman et al., 2004; Rosenberg & Van Hout, 2014). The kappa values were calculated in a second‐by‐second manner in the present study, which reduces the agreement compared with previous studies as most of them evaluated the agreement in epoch‐wise, i.e. typically within in 30‐s epochs (Pittman et al., 2004; Rosenberg & Van Hout, 2014). For example, respiratory events scored by different scorers might be separated by 20 s and be still in the same 30‐s epoch leading to agreement in epoch‐wise analysis but not in second‐wise analysis. Moreover, the agreement values are not directly comparable between the studies as the used dataset (e.g. number of recordings and events) affects the kappa values.

The accuracy of the duration of the scored events was relatively good in all clusters with multiple scorers, i.e. on average the events overlapped with each other by most of their duration. However, it must be noted that the median number of scorers in a cluster was only three (out of 10), and less than half of the clusters were scored by at least five scorers. In other words, there is a high disagreement in what is considered as a respiratory event and what is not but, when in agreement, the start time of the event was defined quite similarly, whereas the end times were more divergent.

One major factor affecting the agreement is the differing interpretations of the AASM rules and possible in‐house scoring conventions, highlighting a need to clarify the respiratory event scoring rules further. For example, in the AASM Manual (Berry et al., 2020; Troester et al., 2023), a rule states that if a portion of an event that would otherwise meet criteria for a hypopnea meets criteria for apnea, the entire event should be scored as an apnea. This could lead to variation at least in the duration scoring but also in the event type scoring. In this study, the highest agreement values were not always found between the scorers from the same sleep centres and thus, the different conventions in centres are unlikely to explain the variability. However, we had only three scorer pairs from the same centres and, therefore, this needs to be investigated in more depth in future studies.

Due to the low agreement, it is evident that changes in the scoring practices are needed. The manual scoring rules need to be updated. Also, training and standardized protocols for assessing and improving the inter‐scorer reliability within centres might be required to reach unified scoring practices. As the lowest agreements were found in central and mixed apneas, hypopneas associated with an arousal, and events during N1 stage, the most efforts should focus on these. Moreover, some scorers scored events shorter than 10 s and hypopneas that were not followed by an arousal or desaturation within a reasonable time after the end of hypopnea. According to the AASM scoring manual (Berry et al., 2020), respiratory events should be at least 10 s in duration, but there is no rule in what time frame an arousal or desaturation should occur for the hypopnea scoring. Events shorter than 10 s were excluded from the analysis as some of them were clearly accidentally scored (less than second long events). Including these events to the analysis was also tested, but it had only a minor effect on the results, for example, Fleiss kappa value for binary analysis did not change. Additionally, excluding hypopneas without an arousal or desaturation did not have major impact on the results as the kappa values changed by 0.02 and the ICCs of AHIs by 0.03 at most. As a recent study reported that the use of computer‐assisted scoring increased the kappa values compared with those resulting from manual scoring only (Alvarez‐Estevez & Rijsman, 2022), the utilization of validated automatic scoring algorithms more often in the future could provide accurate and consistent diagnoses (Alvarez‐Estevez & Rijsman, 2022; Nikkonen et al., 2021; Pittman et al., 2004).

### 4.1 Strengths and limitations

The strength of our study is the in‐depth analysis, which included temporal, i.e. second‐by‐second, agreement of event subtypes and events in different sleep stages, but also the main respiratory event indices and severity classifications. Furthermore, the study included a relatively high number of scorers from multiple sleep centres.

A limitation is that nasal pressure signal in conjunction with the thoracoabdominal belts was used for scoring apneas, which could lead to false apneas in the case of mouth breathing. However, the use of nasal cannula instead of oronasal cannula is unlikely to affect the inter‐scorer agreement as all scorers had the same data to score. Another limitation is that the scorers might have had different conventions when using the scoring software. Furthermore, all scorers were instructed to use automatic scoring prior to manual scoring, and the analysis start and end times differed as the scorers were able to change these time points themselves. Moreover, in a normal clinical setting, the diagnosis is not based solely on the AHI and other indices, but also the symptoms of the patients among other things are considered. In this study, we did not consider the symptoms in the analysis because the aim was to study the agreement based on the respiratory event scoring solely. In addition, we did not consider obstructive or central hypopneas.

Furthermore, the sleep epoch linked to a respiratory event was the epoch during which the event started. Therefore, for some events, part of the event occurred at a different sleep stage than where the event started. The true sleep stage can change during the same epoch due to the low temporal resolution of the hypnogram. However, this same problem is present in all analyses utilizing the standard sleep staging. Moreover, a limitation in the kappa statistics is that the prevalence of the events affects the kappa values (Brennan & Silman, 1992).

## 5 CONCLUSION

In conclusion, the temporal agreement of respiratory events based on kappa statistics was low. The agreement varied depending on the respiratory event type and the sleep stage. The variability in the scoring of respiratory events affected the severity indices and thus, in most of the participants the OSA severity category changed depending on the scorer. This is an alarming finding, as ultimately these differences in the scorings affect treatment decisions. The AASM rules for manual scoring are interpreted differently by expert sleep scorers and lack clarity and thus, improvement and harmonization of the scoring is needed.

## AUTHOR CONTRIBUTIONS

**Minna Pitkänen:** Methodology; software; formal analysis; investigation; visualization; writing – original draft; writing – review and editing. **Henna Pitkänen:** Methodology; software; visualization; writing – review and editing. **Rajdeep Kumar Nath:** Methodology; writing – review and editing. **Sami Nikkonen:** Conceptualization; methodology; data curation; writing – review and editing. **Samu Kainulainen:** Writing – review and editing. **Henri Korkalainen:** Writing – review and editing. **Kristín Anna Ólafsdóttir:** Project administration; writing – review and editing. **Erna Sif Arnardottir:** Funding acquisition; writing – review and editing; project administration. **Sigridur Sigurdardottir:** Data curation; writing – review and editing. **Thomas Penzel:** Funding acquisition; project administration; writing – review and editing. **Francesco Fanfulla:** Funding acquisition; project administration; writing – review and editing. **Ulla Anttalainen:** Funding acquisition; project administration; writing – review and editing. **Tarja Saaresranta:** Funding acquisition; project administration; writing – review and editing. **Ludger Grote:** Funding acquisition; project administration; writing – review and editing. **Jan Hedner:** Funding acquisition; project administration. **Richard Staats:** Funding acquisition; project administration; writing – review and editing. **Juha Töyräs:** Funding acquisition; writing – review and editing. **Timo Leppänen:** Conceptualization; funding acquisition; project administration; writing – review and editing.

## FUNDING INFORMATION

This research was supported by research funding from the European Union's Horizon 2020 research and innovation programme under grant agreement no. 965417, Finnish Cultural Foundation – Central Fund, the State Research Funding for university‐level health research, Kuopio University Hospital, Wellbeing Service County of North Savo (5041794, 5041797, 5041803, 5041804, 5041809, 5041812), the Magnus Ehrnrooth Foundation, and Sigrid Jusélius Foundation (230216). LG was supported by the Swedish Heart and Lung Foundation (20210529) and the ALF Agreement (ALFGBG966283).

## Data Availability

Research data are not shared.
